# Rifted margins classification and forcing parameters

**DOI:** 10.1038/s41598-021-87648-3

**Published:** 2021-04-14

**Authors:** F. Sapin, J.-C. Ringenbach, C. Clerc

**Affiliations:** 1 Total S.A., CSTJF, Avenue Larribau, Pau, France; 2 ISEA, Université de la Nouvelle Calédonie, Avenue Larribau, Nouméa, Nouvelle Calédonie France

**Keywords:** Geodynamics, Geology, Geophysics, Tectonics

## Abstract

Rifted margins are the result of the successful process of thinning and breakup of the continental lithosphere leading to the formation of new oceanic lithosphere. Observations on rifted margins are now integrating an increasing amount of multi-channel seismic data and drilling of several Continent-Ocean Transitions. Based on large scale geometries and domains observed on high-quality multi-channel seismic data, this article proposes a classification reflecting the mechanical behavior of the crust from localized to diffuse deformation (strong/coupled to weak/decoupled mechanical behaviors) and magmatic intensity leading to breakup from magma-rich to magma-poor margins. We illustrate a simple classification based on mechanical behavior and magmatic production with examples of rifted margins. We propose a non-exhaustive list of forcing parameters that can control the initial rifting conditions but also their evolution through time. Therefore, rifted margins are not divided into opposing types, but described as a combination and continuum that can evolve through time and space.

## Introduction

Observations along rifted margins are now more accurate thanks to the increasing number of deep high-quality 2D/3D seismic datasets acquired. They provide images of the deep levels of the crust, its internal geometries, structures and seismic facies, especially within the Continent-Ocean Transition (COT). Coupled with wide-angle seismic and gravity/magnetic data, which give an idea of the velocity/density gradient and layering of the crust, they allow a much better resolution of the large-scale character of a margin. Therefore, the structural diversity of rifted margins is now accessible. From the tilted blocks of the 1970′s, the detachments and mantle exhumation of the 1980′s–1990′s to the great variety of geometries now seen^1^, we revisit the classification of these margins.

The extensional models of McKenzie^2^ and Wernicke^3^ were developed based on observations from onshore and nearshore rifted basins. They were extrapolated to the entire margin and considered as references for the crustal thinning. Breakup was supposed to occur when the crustal stretching factor was reaching the value of 5 for a 30 km thick continental crust^2,4^, allowing magma to suddenly breach through the crust^5^.

In the late 1980′s and 1990′s, an increasing collection of data (multi-channel seismic, dredges and drillings) in the distal part of the Iberia-Newfoundland conjugate margins provided a better picture of the COT^6^. In addition, alpine geologists highlighted the convergence with geological features observed in the Swiss Alps and developed original models integrating the complete evolution from the first increment of continental extension to oceanisation^7–10^. From these combined studies arose the magma-poor model of rifted margins, characterized by limited volumes of syn-rift magmatic rocks and exhumation of continental lithospheric mantle in the COT^11–13^.

In the meantime, other data collected offshore^14–17^ and onshore^18,19^ in the Northern Atlantic led to the development of a model for Volcanic, or magma-rich, Rifted Margins (VRM)^20,21^. These margins are characterized by thick wedges, up to 20 km, of Seaward Dipping Reflectors (SDR)^22,23^ mainly composed of basaltic flows interbedded with sediments and paleo-soils, all intruded by sills. VRM also possess overall high velocities (6–7 km/s) and High Velocity Lower Crustal Bodies (HVLCB; 7.2–7.5 km/s)^24,25^. However, the different observations did not allow sufficient convergence of ideas to reach a consensual model^26–30^.

These spectacular observations tend to polarize rifted margins interpretation towards two opposite types based only on magmatic productivity.

Since the 2000′s, many numerical models tried to test different physical parameters, especially the rheological strength of the crust^31–37^. They recently allowed to explore these parameters in 3D^38–40^. In parallel, seismic profiles from several rifted margins revealed structures not predicted by neither the magma-poor nor the magma-rich models^27,41,42^. In agreement with previous numerical modelling results^35,36^, some authors proposed the existence of at least a third type of rifted margin, characterized by the inability of the deformation to localize until magmatic breakup occurs in a highly stretched crust.

As a consequence, the mechanical behavior of the margin, that were not generally discussed in the magma-poor/magma-rich classification, became another axis for rifted margins description. Two endmembers were described (weak and strong mechanical behaviors) but, thanks to thermomechanical modelling, a wide spectrum of intermediate behaviors was explored.

**Figure 1:**
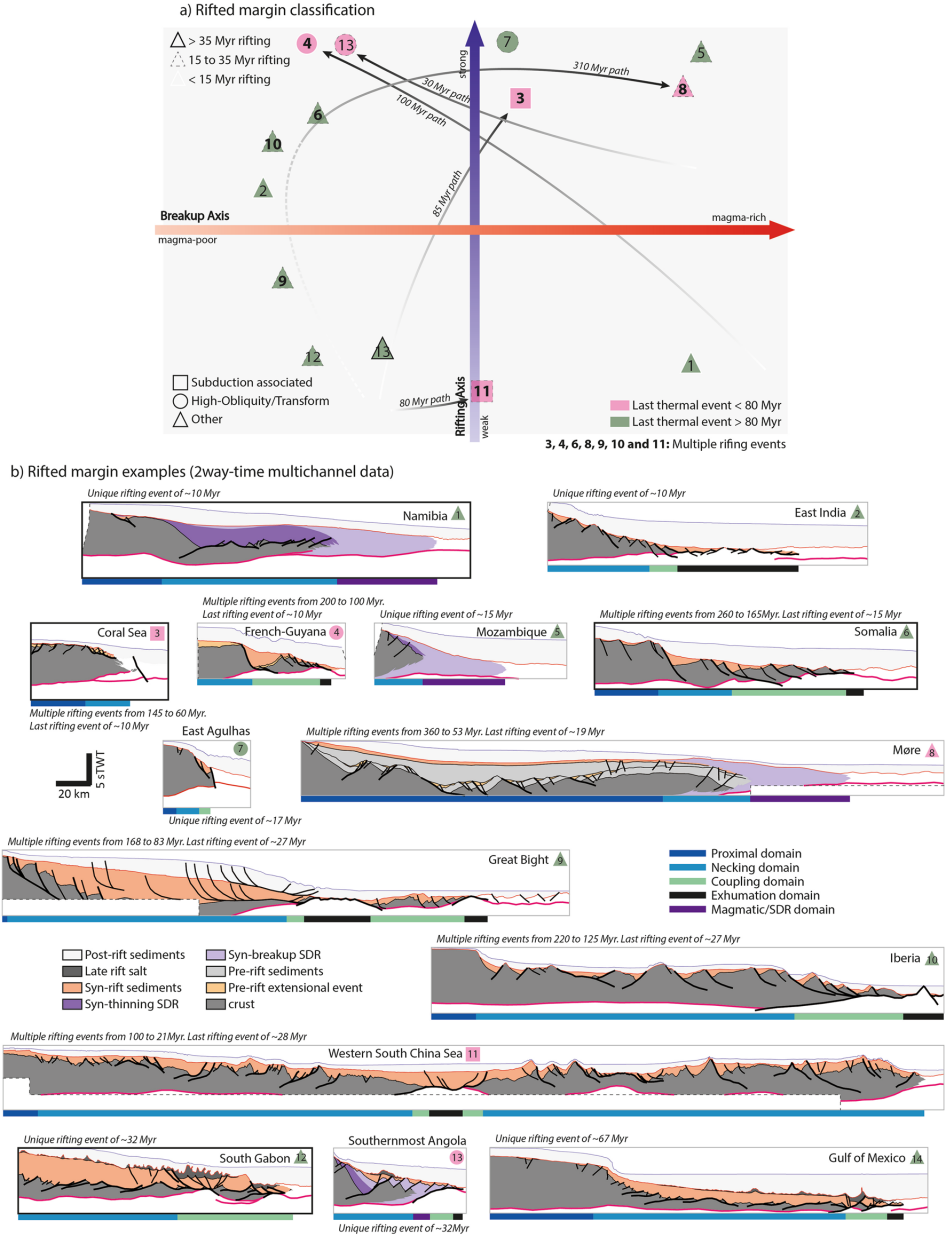
Classification of rifted margins (**a**) and some examples ranked by rifting duration (**b**). The proposed classification is organized along two axes, the rifting axis that characterizes the overall rheology of the crust and the breakup axis that characterizes the quantity of magma involved during the rupture of the lithosphere. The four presented examples, Somalia (6), South Gabon (12), Namibia (1) and Coral Sea (3), are highlighted in a thick black frame. The other sections are either from published work: Great Bight (7)^129^, East India (8)^103,130^, French Guyana (9)^131^, Iberia (11)^13^, Møre (14)^132^; or from Total internal studies: Southernmost Angola (5, Namibe Basin), Western South China Sea (6), Gulf of Mexico (10, Mexico), Mozambique (12) and East Agulhas (13, South Africa). All lines are located in Fig. 2.

Based on the interpretation of deep seismic datasets across many margins and the existing knowledge, we present a classification based on two axes (Fig. 1): Mechanical Behavior, reflecting the deformation style of the crust (weak to strong mechanical behavior) and magmatic production, reflecting the quantity of magma involved during rifting and breakup (magma-poor to magma-rich).

**Figure 2:**
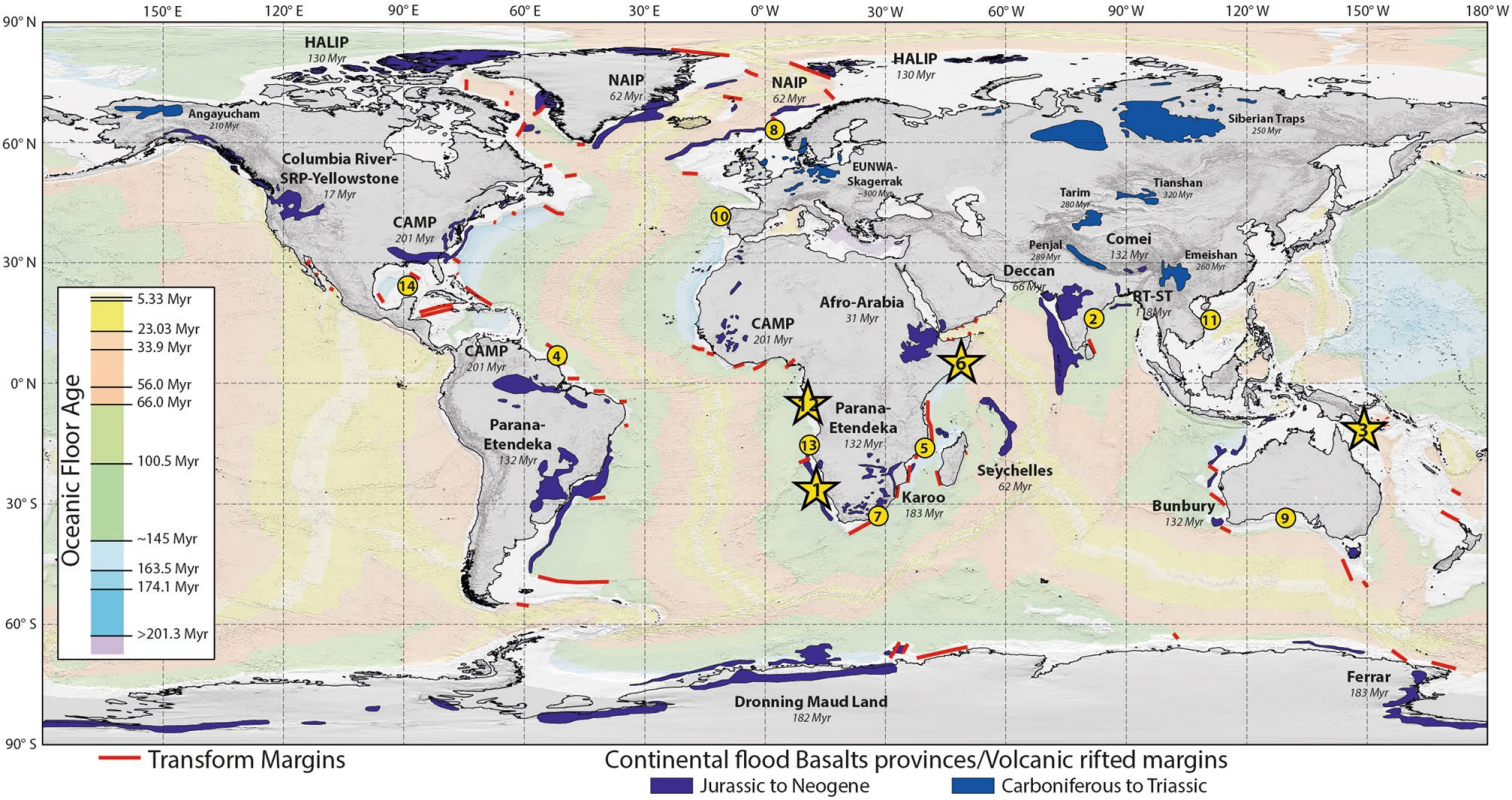
Location of the examples and case studies. The examples shown in Fig. 1 are located on the world map with the oceanic floor age from Müller et al.^133^. The main case studies are emphasized with stars. The main transform margins^134^ and continental flood basalts provinces/volcanic rifted margins ( modified from Bryan and Ferrari^135^) are also shown.

The main seismic characters, geometries and large-scale correlations are illustrated through four contrasting cases located along the Somalia, Uruguay, Gabon and Coral Sea margins (Fig. 2). After describing their structural variability, we discuss this classification and the lithospheric processes involved.

## Archetypal rifted margins examples

The four presented sections illustrate striking geometries and large-scale organizations. Their description follows the methodology described in the Method and Terminology section.

### Localized deformation, narrow necking and late magmatic breakup: Somalia

**Figure 3:**
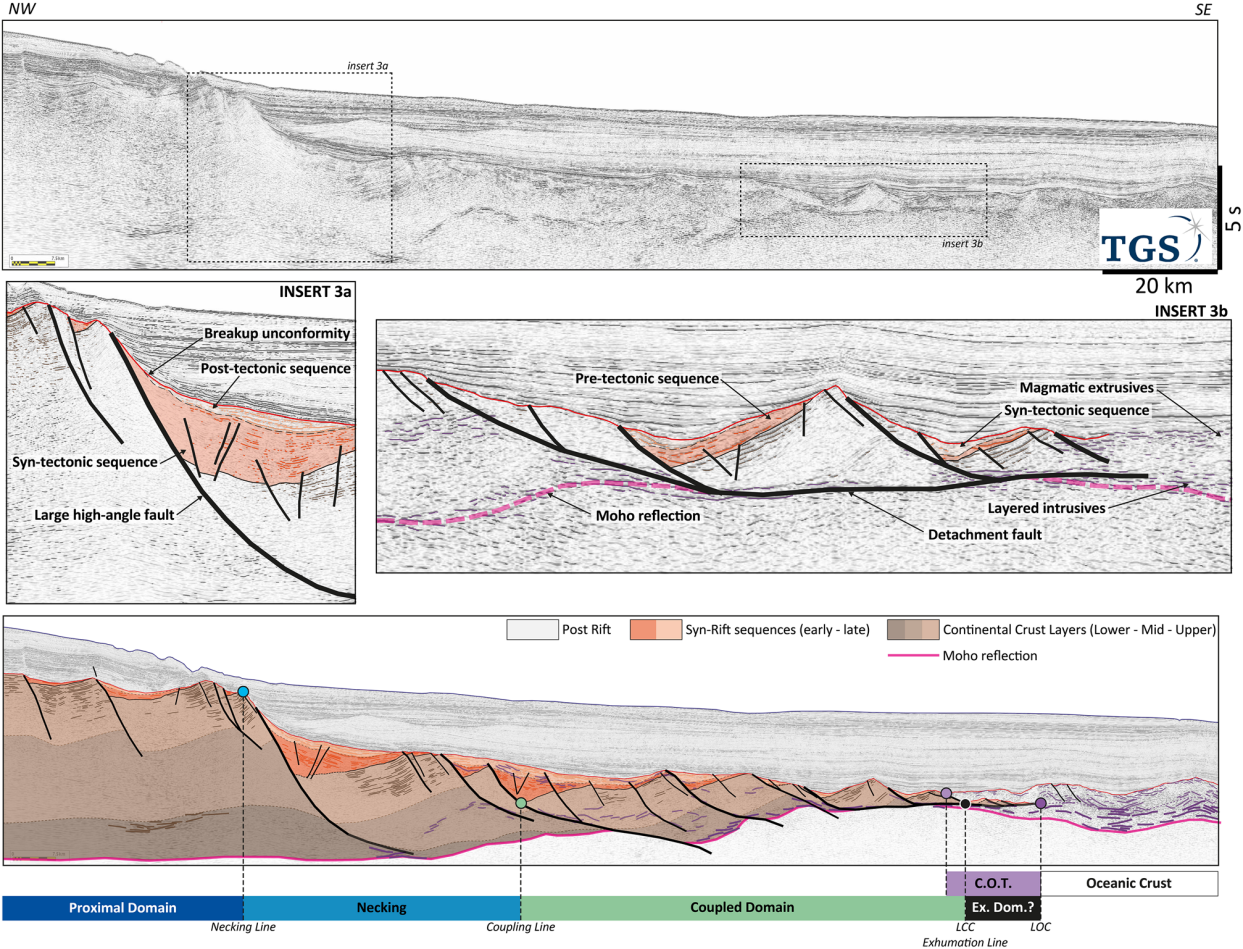
Somalia case study. The section is characterized by a short necking domain accommodated on a couple of high-angle normal faults. The coupled domain is longer with faults extending down to the mantle and cutting the interpreted Moho reflection in several cases. The most external domain is characterized by low-angle faults and detachments exhuming the mantle and leaving rafts of the pre-rift material on the exhumation surface. The emplacement of oceanic magmatism is progressive over this exhumed mantle. The data are courtesy of TGS.

The Eastern Somalia margin formed during the Middle Jurassic as the result of southward separation of the Madagascar-Seychelles-India block from Africa^43^. It displays only limited rift-related volcanism^44^ and developed in a context of carbonate sedimentation with little clastic input. We present a 210 km long and 12 s deep profile (Fig. 3) from TGS.

In the proximal domain of this margin (Fig. 3) three layers are distinct in the basement. The upper layer represents a bedded sedimentary facies. The middle layer is transparent with very few visible structures. The lower layer exhibits high-amplitude, layered facies typical of the lower crust.

The upper layer, interpreted as sedimentary pre-rift, is up to 6 km thick (2.5 sTWT), the transparent upper crust (middle layer) is ~ 15 km thick while the lower layer is thin (~ 8 km) based on Pre-Stack Depth Migration seismic data. The inherited East African crust is said to be of Mesoproterozoic gneisses and meta-sediments reworked during the Panafrican Orogeny^43,45^ and covered by a large Karoo sag basin.

The Proximal Domain underwent modest stretching. The brittle upper crust is cut by high-angle normal faults, bounding half-grabens with limited syn-rift strata packages. The Necking Domain is narrow (~ 50 km; Fig. 3). It is characterized by a top basement and a seismic Moho converging oceanward, indicating an effective thinning of the continental crust. This thinning is accommodated along a couple of high-angle normal faults that seem to root within the lower layer of the continental crust, nearly at its base. The Necking Line is defined by the footwall cut of this first large trans-crustal fault, coupling down to the mantle. The associated sedimentary infill is thin (sediment-starved) with two sequences (Fig. 3 insert 3a): a syn-tectonic sequence with sediment wedging towards the fault and a post-tectonic package which corresponds to a local sag, which tilted later.

The Coupling Domain presents several low-angle normal faults merging into a single detachment (both Limit of Continental Crust (LCC) and Exhumation Line (EL), Fig. 2). This domain is characterized by a wide (~ 75 km) secondary necking zone and a decreasing size of the tilted blocks. The syn-rift infill is thin (Fig. 3 insert 3b).

Seaward of the LCC, a flat, high-amplitude level is interpreted as a shallow top mantle below triangular blocks of hyper-extended crust. The mantle is almost exhumed. It appears likely that in this area, rider blocks of continental crust, similar to extensional allochthons, as defined by Manatschal^10^ in the Alps, are imaged. Both these blocks and exhumed mantle are further seaward covered by magmatic additions, an area interpreted as forming the COT. The COT corresponds to the onset of minor magmatic features (volcanic mounds, sills, underplating) increase in frequency and size seaward and evolve finally towards a thin proto-oceanic crust prior to a 2 s (TWT) oceanic crust (Fig. 3). The edge of the oceanic crust is marked by an outer-high which is onlapped at the top by the breakup unconformity (Top Rift).

### Long necking taper, multiple core complexes and late magmatic breakup: South Gabon

**Figure 4:**
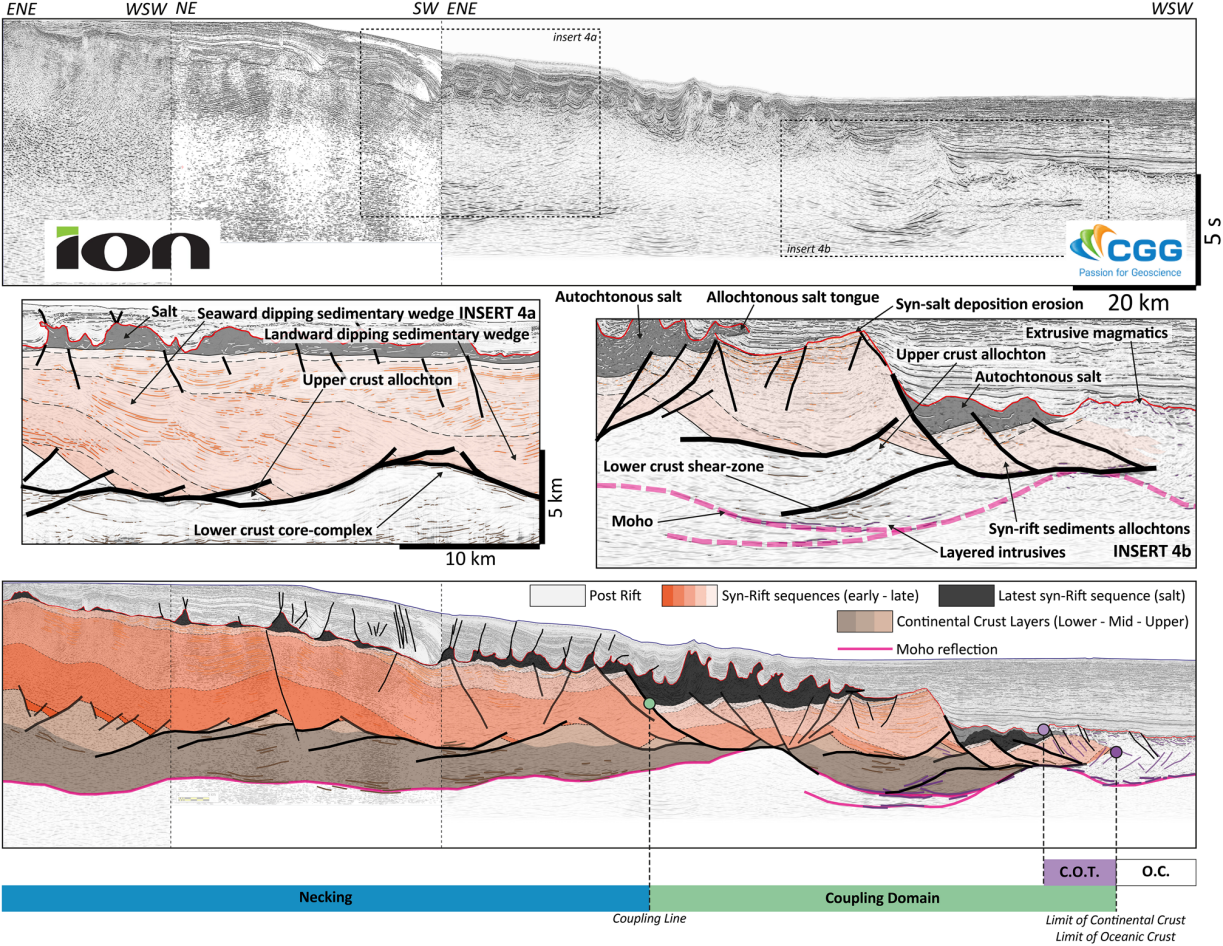
South Gabon case study. The section is characterized by a very long necking domain with a primary necking and a long low angle wedge toward the distal domain. The thinning of the crust is accommodated on low angle normal faults and core-complexes. An important continental to deltaic sedimentation (10–12 km) is associated to this long thinning of the crust with seaward prograding sequences. The coupling domain is marked by younger high-angle faults creating important topography at the base of the salt and cutting through the previous large sediment package and the lower crustal bodies. Local basins with mantle exhumation may form prior to the generation of the oceanic crust. The data are courtesy of ION and CGG-MCNV, all rights reserved.

The Gabon and Brazilian conjugate margins broke up in the Early Cretaceous (133 to 115 Ma)^46^. We present a 195 km long and 12 s deep composite line from the Southern Gabon Margin (Fig. 4) acquired by ION (25 first kms) and CGG (remaining 170 kms of a Fast-Track PSTM).

Although seismic imaging of the syn-rift and basement is limited due to very irregular post-rift sedimentary deposits, resulting from salt tectonics on the Latest Aptian salt layer, the continental crust appear stretched over more than 150 km (Fig. 4). Thus, the Proximal Domain is mainly preserved in onshore and is not present in the area covered by the seismic line.

The Necking Line is close to the coast and the Necking Domain is wide, extending over more than 110 km across the shelf. The thinning of the crust is accommodated along a series of dominantly landward dipping low-angle normal faults and detachments with local core-complexes exhuming lower crustal material^27^. The necking is associated to an up to 12 km thick syn-rift sequence of continental/lacustrine sediments (sampled in several pre-salt distal wells) wedging towards the detachment faults and the core-complexes (Fig. 4, insert 4a). Within the crustal layer, several strong sub-horizontal reflectors are interpreted as crustal shear zones bounding upper crustal boudins on top of lower crust^27^. This pattern evokes semi-brittle anastomosed shear-zones as identified offshore Britain by Reston^47^ and modeled by Jammes et al.^48^ and Theunissen et al.^49^. The sedimentary wedges young oceanward indicating a seaward migration of the deformation^27^ and/or of the deltaic system overfilling the system.

The distal domain presents younger large high-angle normal faults cutting down to the mantle and dissecting the entire pre-existing syn-tectonic sequence and the crustal layers. This inversion of the dip angle and dip direction of the normal faults attests to a change in the mechanical behavior of the distal margin. This change may have occurred in response to the coupling of the hyper-thinned continental crust leading to a shift from ductile to fragile behavior. This shift is dated Mid to early Late Aptian (AP2 surface penetrated by several wells in the area) and created local grabens with salt^50^ preserved in the most distal domain (Fig. 4, insert 4b).

This line does not cover the large exhumation domain that, however, it is present further south together with increasing magmatism^50^.

The COT corresponds to the onset of magmatism (volcanic mounds, sills, underplating)^50^ that intrudes the faulted late-rift sediments and evolves to a thin oceanic crust prior to a 2 s (TWT) thick mature oceanic crust (Fig. 4).

### Long necking taper and early magmatic input: South Namibia

**Figure 5:**
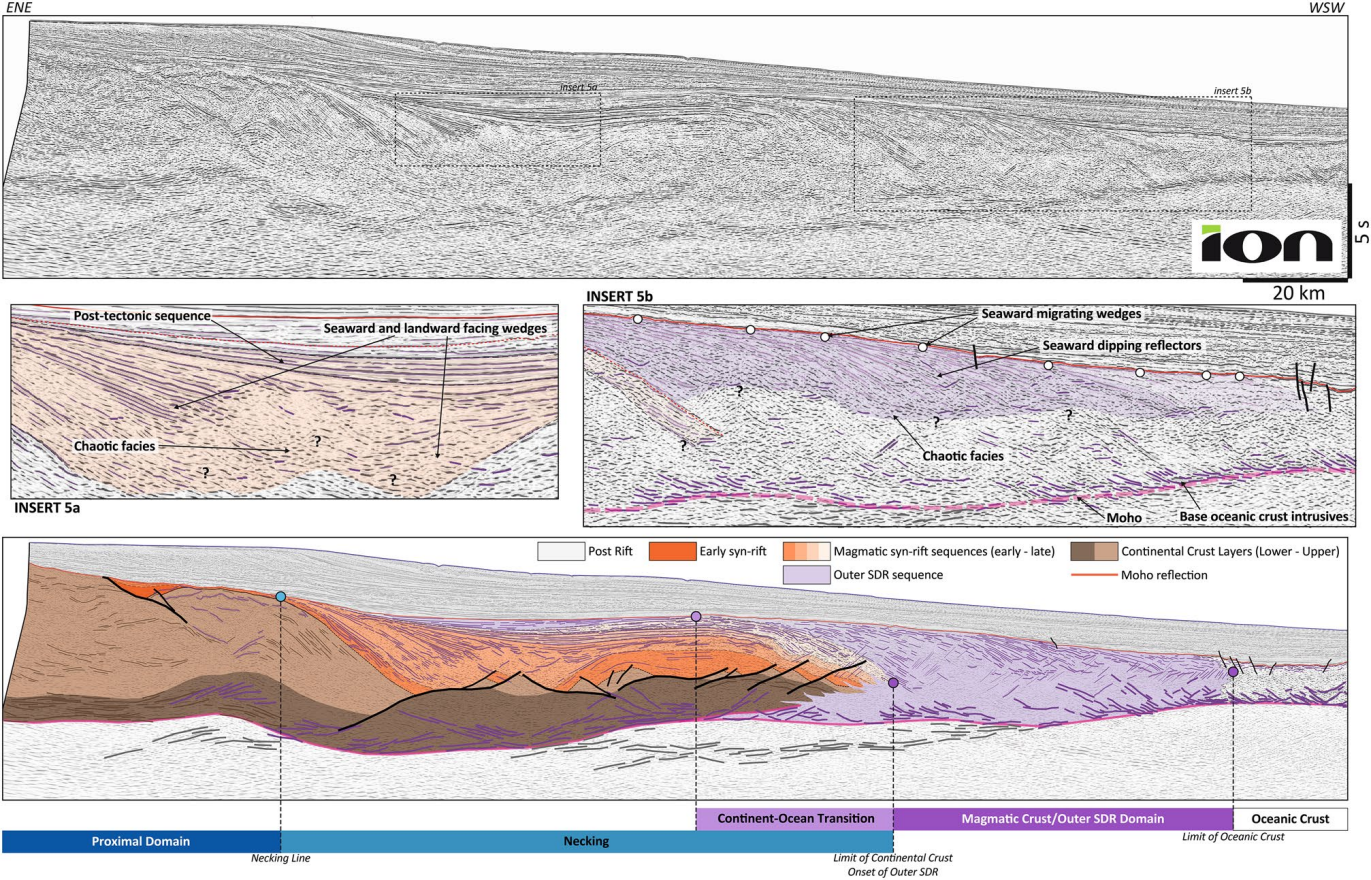
South Namibia case study. The section is characterized by thick syn-rift wedges of Seaward Dipping Reflectors (SDR)^30,63,136^. The thinning of the crust seems to be accommodated on low angle normal faults and core-complexes but strongly intruded by multiple magmatic features (dikes, sills). It ends up with a very long necking domain with a primary necking and a long low-angle wedge towards the distal domain. A large basin with both Seaward and Landward Dipping Reflectors develops in continuity with the primary necking. Their relationship with faults and potential core-complexes are unclear^30^. There is no coupling domain, the most distal domains are replaced by thick SDR packages thinning toward a classic oceanic crust. The data are courtesy of ION, all rights reserved.

The Namibia-Uruguay VRM results from the breakup of Gondwana^51,52^, which is at least partly contemporaneous with the emplacement of the Parana-Etendeka large igneous province^53^. The austral South Atlantic Ocean opened across two generations of orogenic belts^54,55^: the Late Paleozoic Ventania-Cape Fold Belt to the south and the Pan-African/Brazilian Ediacaran orogenic belts to the north. We present a 255 km long and 14 s deep line from the South Namibia Margin (Fig. 5) acquired by ION.

The most striking structure of all these conjugate margins are SDR wedges (Fig. 5). They were emplaced between the oceanic anomalies M10-M11 (135 Ma) in the south^56^ and M2 (128 Ma) in the north^57^.

The Proximal Domain exhibits two crustal layers (Fig. 5). The upper crustal layer shows well-bedded reflections organized in large folds and nappe-shaped structures. They might be inherited from the previous orogens^55^. Small normal faults rooting in internal levels of this upper crust are bounding half-grabens filled by upper Lower Cretaceous sediments. The lower crustal layer shows the layered, high-amplitude seismic facies diagnostic of the lower crust.

The Necking Line corresponds to the onset of the SDR facies in association with a deepening of the Top-Basement/Base Rift horizon. The primary necking is rather short but followed by a wide crustal taper (> 100 km). The crustal layering is difficult to observe but the presence of both Seaward and Landward Dipping Reflectors (Fig. 5, insert 5a)^30^ and high-amplitude features cutting the lower crustal levels suggests a strongly intruded crust and possible mafic underplating. This lower crustal layer presents highly reflective sigmoidal reflectors interpreted as sheared structure.

The distal domain, or Magmatic Domain here, is characterized by the high-reflectivity SDR wedges. The identified Moho and the top of SDR converge towards the oceanic crust (distal necking)^30^. Internally, this domain exhibits three layers (Fig. 4, insert 4b):

- The upper unit is characterized by large flat-lying high-amplitude reflectors, the SDR. They are well bedded and organized as a series of wedges deposited in-sequence oceanward^30^. The size of these wedges is decreasing oceanward and their curvature increases accordingly^30^. The most distal SDR present an increasingly more chaotic facies, suggesting a different mode of emplacement. In refraction seismic data^58^, these facies are characterized by a gradient of velocity from 5 to 6 km/s in the inner wedge and 6 to 6.3 km/s in the outer wedge, close to the velocity of the middle unit;
- The middle unit is distinct from its chaotic facies. Its top, corresponding to the downward tip of the SDR, is not a clear horizon but of very irregular shape. This shape might be caused by sill intrusions and some more vertical features, nearly perpendicular to the SDR, that can be interpreted as sheeted dikes. The base of this unit is also irregular and not easily observable;
- The lower unit is composed of an increasing amount of high-amplitude flat-lying or both landward and oceanward dipping features. Closer to the Moho the high-amplitude reflections are increasingly flatter. Geophysically, the middle and lower units are part of the same velocity/density layer^58–60^. Their velocity structure is globally homogenous with a velocity between 6.8 and 7.1 km/s. This velocity is similar to that of ductile lower crust below the inner SDR^58,61,62^, but also similar to the lower unit of the oceanic crust in the area^58,59^. These seismic facies and velocities tend to support that the crust below the outer SDR is dominantly to entirely magmatic^28,30,63,64^.

### Core complexes, short necking and magmatic breakup: North Coral Sea

**Figure 6:**
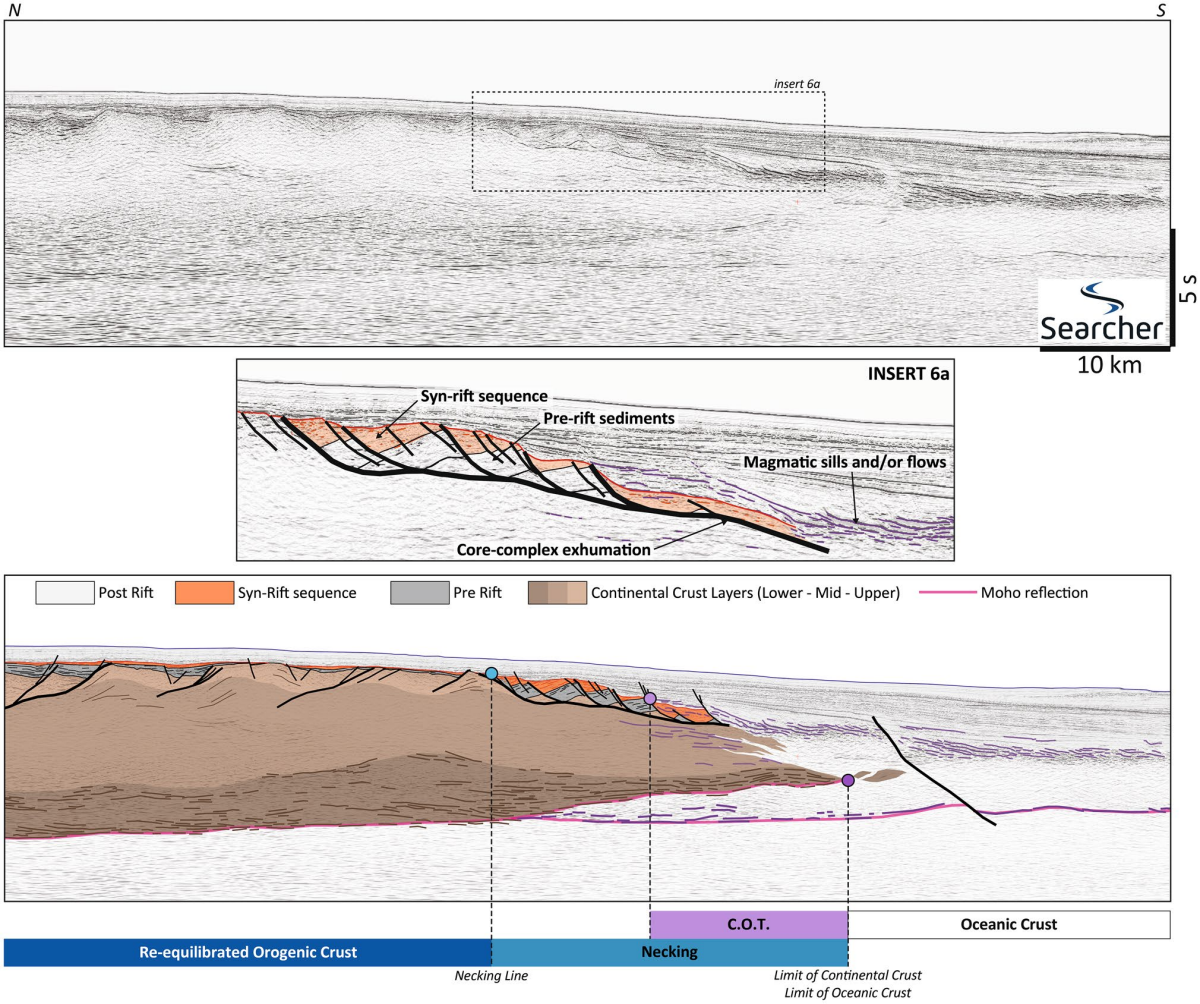
North Coral Sea case study. The section crosses a proximal domain in a continental crust already thinned (23–24 km). The final thinning of the crust is expressed on a single shallow detachment and important shear structures in the lowermost crustal layer. The necking zone is short. Breakup is rapid with short OCT leading to a classic oceanic crust in terms of thickness but with a peculiar upper layer made of interactions between magma (intrusive and effusive) and locally high sedimentation. The data are courtesy of Searcher.

The Coral Sea opened as a marginal basin above the East Australian retreating slab in a context of post-orogenic collapse^65,66^. Locally, two basins are involved with a Late Cretaceous rifting for the subducted/obducted Emo Basin^67^ and Latest Cretaceous/Paleocene for the younger Coral Sea Basin. We present a 90 km long and 14 s deep line through the northern margin of the Coral Sea (Fig. 6) acquired by Searcher.

The continental crust is 23–24 km at the thickest. The lower crust is strongly reflective, with high-amplitude continuous and curved reflectors drawing sigmoidal patterns typical of crustal shear zones. The middle crustal facies is chaotic, and the upper facies presents some tilted bedding attributed to the previous orogeny and/or the slightly older Emo rift^68,69^. On other profiles from our dataset, the upper continental basement of the margin shows indications of a pre-existing fold-and-thrust belt, with some thrusts inverted as low-angle detachments.

Another striking feature is the scarcity of syn-rift deposits with some of them being even eroded before the deposition of post-tectonic sediments. Such patterns can be explained through a core-complex model in a thermally-supported setting (e.g. Basin-and-Range province^70^) involving progressive uplift of the detachment fault and of its rider blocks through a rolling hinge mechanism^71–73^. Several of these core-complexes and short normal faults rooting at the interface between the upper and middle crustal layers are visible along the profile. However, large scale correlations attribute these structures to the previous orogenic collapse^74^.

The narrow Necking Domain exhibits a thinning of the crust from 22 km down to a 7–8 km thick and is in direct contact with the steady oceanic crust of similar thickness. The Necking line is marked by a single detachment decoupling at very shallow crustal levels (3–4 km) and rafting away elements of pre-rift sediments and upper crust (Fig. 6, insert 6a). This detachment evolves laterally into a more typical metamorphic core-complex exhuming middle and lower crustal levels covered and partly masked by COT volcanism. The infill of the rafted blocks by syn-rift sediments is limited (Fig. 6, insert 6a).

The COT is marked at depth by a sudden rise of a first deep and coherent reflector. A second, deeper and flatter reflector, is interpreted as the post-breakup Moho below underplated and/or strongly intruded lower crustal material. Towards the surface, this transition corresponds to an abundance of strong and discontinuous reflectors, that contrast with the surrounding sediments and underlying basement.

The oceanic crust presents a peculiar upper facies (Fig. 6), with high amplitude features interbedded and/or intruding the post-rift sediments and dipping seaward. These features are interpreted as sills or lava flows. They might be linked to the interaction between sediments and magmas at the ridge during the early accretion as observed for example in the Andaman Sea^75^.

## Rifted margins classification

The distinction between magma-rich and magma-poor margins is mainly a matter of volume of magma involved from rifting to breakup. A complete spectrum may exist between these two types.

Furthermore, numerical modelers define two other categories, weak and strong, based on the content of ductile material within the continental crust. Even if ductile deformation is known from the Basin-and-Range world-class analog^70^, potential fields analogs for fully developed weak crust margins are rare and debated^76,77^.

The general and simple magma-rich/poor combined with the weak/strong crust classification can be efficiently used to classify the world’s rifted margins (Fig. 1a):

- The Rifting Axis considers the mechanical behavior of the crust while rifting. Two poles are opposed: weak (diffuse/decoupled deformation) and strong (localized/coupled deformation) mechanical behaviors;
- The Breakup Axis considers the amount of magma involved from rifting to breakup, from a magma-poor to a magma-rich pole.

### Rifting axis

A seismic lines shows the final structure and addressing the mechanical behavior of the rifted crust is uneasy as it results from the combination of several parameters that vary through time (extension rate, crust and mantle composition, thermal state, sedimentary forcing, initial thickness, age and lithology of the crust, inheritance, etc.).

The strong pole is well known as it generates rather typical rifting structures. Its geometrical characteristics are:

- A short Necking Domain in which the deformation is accommodated on very few structures;
- High-angle normal faults in the proximal margin, evolving in the Necking Domain into large listric faults rooted in the middle to lower crustal layer (4, 5, 6 on Fig. 1);
- When existing, the proximal Coupling Domain is thick as supposedly, mostly the brittle layer(s) of the crust are preserved^78^. Thus, the stronger the crust is, the thicker and longer will be the coupled domain as it will take several faults to thin this domain prior to breakup^79^.
  The weak mechanical behavior have been widely explored by numerical modeling^35,80–82^. On seismic, we can identify several geometrical characteristics that tend to suggest the existence of this type of behavior:
- A proximal and sharp onset of necking followed by a wide crustal taper (1, 11, 12, 14 on Fig. 1). This crustal taper, as suggested by thermomechanical models, is formed by a spreading of the continental crust over large distance before coupling and/or breakup^35,49,82^. This wide Necking Domain is explained by the inability of the deformation to localize and couple with the mantle;
- Other geometries suggesting ductile deformation can be interpreted on seismic at the base of the crust along rifted margins both in magma-poor or magma-rich settings (1, 3, 11, 12 on Fig. 1). Along the South Gabon margin for example, 10 km to 50 km-long crustal-scale lenses are observed (Fig. 4)^27^. The base of the boudins, at a depth of 15 km to 12 km is separated from the Moho (17 km to 15 km-depth) by a 3 km to 5 km-thick lower crust characterized by long and sub-horizontal reflectors. This lower crustal layer is particularly evocative of a ductile mechanical behavior, especially in the inter-boudin necks where it rises/bulges in between more resistant (upper/mid) crustal boudins. Similar structures^83^ can also be observed in magma-rich settings (Fig. 5)^27,30^;
- A shallow level of rooting (6 – 8 km deep) and the presence of numerous low-angle normal faults is also a characteristic of the deformation associated to weak behavior (3, 11 on Fig. 1).

### Breakup axis

This axis opposes the magma-poor and magma-rich poles. These two extremes are well described with numerous data and outcrop analogs. The recent ODP campaign on the South China margin^84,85^ evidenced an intermediate case, confirming old concepts primarily invoked for continental lithosphere breakup in the 1970′s^5^ relying on observations and physical measurements of oceanic ridges^86–88^. Therefore, reintroducing a certain variability along this axis is mandatory. Indeed, this axis reflects the ability of the mantle to tear the lithosphere apart to generate a new oceanic crust. This magmatic input is also often correlative to the timing of breakup. Likely, the more magma, the earlier and more sudden the lithospheric breakup will be. The definition and key observables between these two poles are numerous and focus mainly onto the more distal domains.

The magma-rich pole is reached when continental extension is coeval with the production of large quantities of melt from the mantle. Its characteristic geometries are SDR^16^:

- Inner SDR develop during the rifting and thinning of the crust^21^ and their bounding faults seem to die out along the top of the middle to lower crust^27,30^. Outcrops from the north Scandinavian Caledonian VRM show that this lower crust is heavily sill-injected^89^ and sheared^27,30^. The intervening faults are often injected by magma. Through our examples (Fig. 1b), the inner SDR Domain, correlative to the Necking Domain, can be of variable width, and can even be almost absent;
- The key characteristic of magma-rich margin is the outer SDR domain, or Magmatic Domain. Its presence defines the magma-rich pole. Its width seems to be homogeneous (50 – 60 km; 1, 5, 8 on Fig. 1). It always exhibits the same triangle shape in which the top of the SDR and the Moho reflection are converging seaward (Distal Necking)^30^. Outer SDR are also associated with linear magnetic anomalies^90,91^. They reveal that the formation of this magmatic crust may be rapid at rift scale (1 to 3 Myr).

On the other hand, magma-poor margins form when continental extension is not coeval with large magmatic production. The lithospheric extension must be accommodated by tectonic structures. This process has often been compared to very slow spreading ridges^92^ where the velocity of extension is so slow that mantle decompression is not fast enough to produce melt. Serpentinized lherzolitic mantle is brought to the surface by tectonic processes^93–95^. The generation of the distal margin, in the absence of magma, leads to the formation of two sub-domains: Coupling Domain and Exhumation Domain. In extremely magma-poor margins, both domains are present, and the Exhumation Domain can reach a width of more than 150 km (2, 9, 10 on Fig. 1). The Exhumation Domain represents a transition zone located between the edge of the continental crust (LCC) and the first unambiguous oceanic crust (LOC). It is mostly composed of exhumed serpentinized continental lithospheric mantle and few magmatic mounds^6,96–100^. This is further supported by reflection and refraction data^101,102^ or potential field data^103^. The transition towards a steady-state oceanic spreading it still poorly known but seems to be sharp (Figs. 3 and 4) to continuous in extremely magma-poor systems^104^.

## Discussion

**Figure 7:**
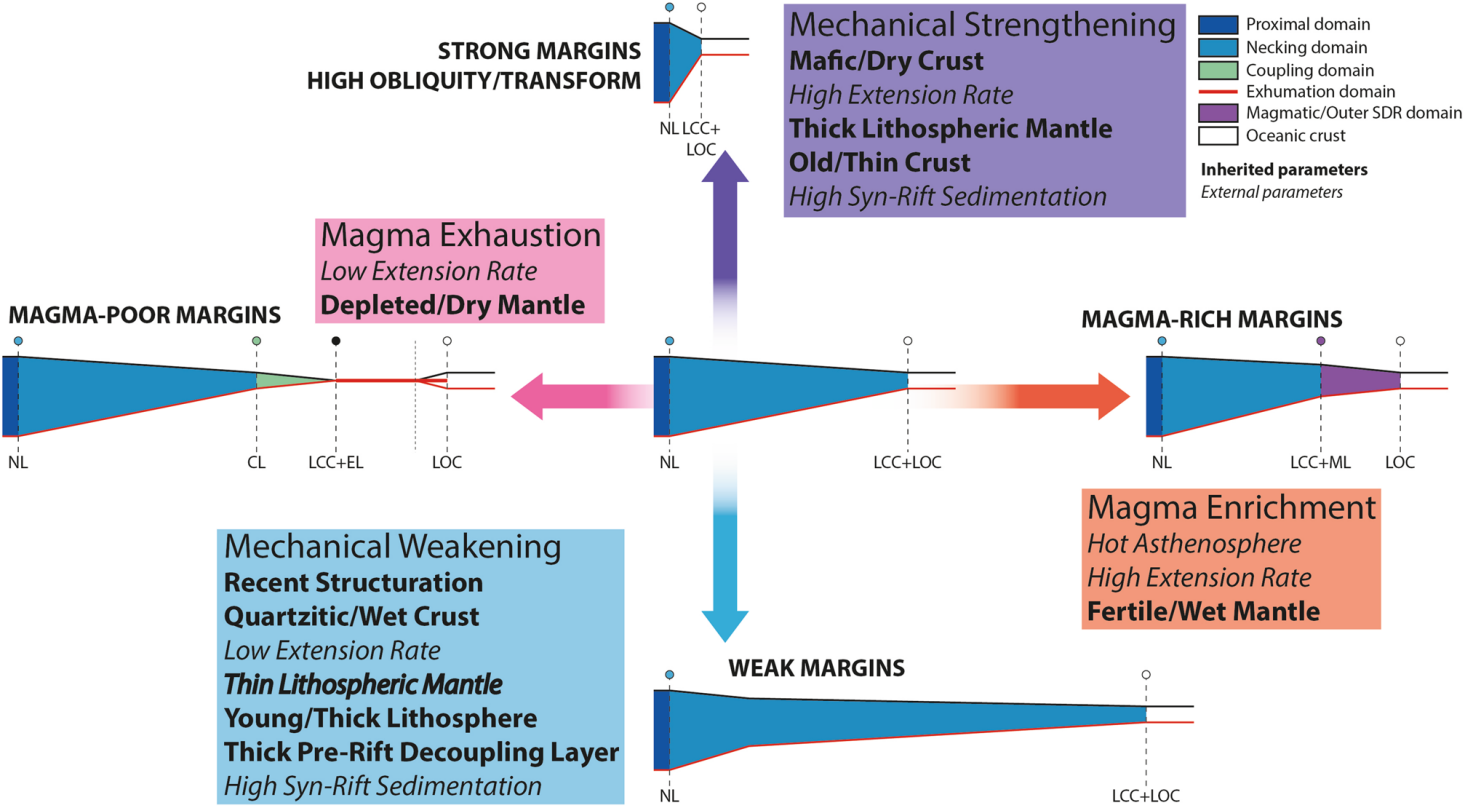
Forcing parameters and their influence on Rifting and Breakup axes. These parameters may be either inherited or external. When external, they can change the behavior of the margin drastically at any stage of the rifting. The obliquity is a key parameter as it seems to overprint any initial rheological/magmatic conditions. NL: Necking Line, LCC: Limit of Continental Crust, LOC: Limit of Oceanic Crust, CL: Coupling Line, EL: Exhumation Line; ML: Magmatic Line.

Crustal mechanical behavior and mantle melting capacity are controlled by the interplay of several parameters, enhancing or lessening each other. They can even evolve while rifting. The listed parameters here below are seen either as inherited or external. They are likely non-exhaustive (Fig. 7) and sorted to show their effect on either the rheology, or the mantle melting capacity, or both.

### Obliquity

The obliquity of the margin is defined as the angle between the principal trends of the margin (especially COB/LOC) and the involved plates motion. Most of the world’s rifted margins present a certain degree of obliquity. Indeed, modeling suggests that oblique extension facilitates rifting and continental breakup^106^. Most extreme cases of obliquity (transform and high-obliquity margins) represent about 31% of the rifted margins^107^. These types of segments can be perceived as either inherited (reactivation of basement lineaments^107^) or newly formed (link between two divergent segments^39^).

From the architecture, transform and high-obliquity margins occupied the strong position of the proposed classification (Fig. 1). Indeed, the associated structures are so steep that they are able to couple the different levels of the lithosphere. Thus, obliquity seems to polarize the margin architecture toward the strong behavior pole, even locally, independently of the global rheology of the crust.

### Inherited parameters

The inherited parameters are sorted in order of decreasing impact on the mechanical behavior of the crust and magmatic production of the mantle:

- Crust and mantle composition: the importance of the crustal chemical composition on the strength profile of the crust is known since several decades^108–110^. With simple compositions (Quarzitic, Felsic, Mafic, etc.), the wetness of the mineral assemblage will also influence the strength of the crust. In thermomechanical modeling, more complex compositions can be set up by multiplying layers of distinct mineral assemblage. The case of a mafic lower crust is often used leading to a general strengthening^31^. The composition, temperature and wetness of the lithospheric mantle will influence the magmatic productivity of the mantle. The richness in fertile elements and a higher content in water tend to favorize a high melt production^111–114^;
- Lithospheric thickness: this parameter represents the pre-rift thermal structure. In nature, little information is available on the geotherm before the last rifting event initiates. It is a function of the radiogenic heat production and the crustal/lithospheric thickness^115^. The lithospheric thickness increases with the age of the lithosphere (from Phanerozoic to Archean). Artemieva^116^ proposed a linear relation between age and thermal state of continental lithosphere. It is thus important to know when the last thermal event prior to the final rifting affected the future margin and its eventual intensity;
- Thickness and age of the crust: crustal thermal state depends on the crustal age and crustal thickness^116–118^ as both parameters will influence the radiogenic heat production. In a simple manner, the younger and thicker the crust is, the larger its temperature gradient will be;
- Inherited structures may have either a positive or negative influence on mechanical behavior. Indeed, pre-existing faults (strike-slip zone, narrow rift, etc.) will help in localizing the rifting if optimally oriented^119^, giving the apparent effect of a stronger crust (fast coupling). In an opposite way, large deformed areas with important layering of the crust and numerous heterogeneities such as suture zones or arc settings may induce a more diffuse deformation and introduce several internal decoupling layers within the crust, weakening it^119^.

### External parameters

The following parameters are also sorted in order of decreasing impact on the mechanical behavior of the crust and magmatic production of the mantle. These parameters can impact before and during the formation of the rifted margin:

- A mantle plume has two main effects. Firstly, the base lithosphere is hotter than usual due to deep mantle upwelling and can reach values over 1500 °C (Iceland^120^). This induces an increase of the partial melting rate within the mantle leading to a higher magma production. Secondly, it thins the lithospheric mantle through a rise of the asthenospheric mantle, heating the Moho. This in turn will increase the geothermal gradient and may weaken the crust ^117^;
- Extension rate: in agreement with their numerical models, Brune et al.^36^ indicate that the width of the conjugate Central South Atlantic margins increases with the extensional velocity. Oppositely, Huismans & Beaumont^32,33^ showed that high rift velocities strengthen the viscous parts of the crust resulting in a stronger coupling between the upper crust and the lithospheric mantle forming narrow margins. At lower extension rates (< 1 cm/yr), which is generally the case during rifting, the rheology of the lithosphere is of primary importance^32,33,105^;
- High syn-rift sedimentation: surface processes promote the localization of the plastic deformation due to the reduction in topographic and flexural forces that oppose fault displacement^121^. High erosion and sedimentation rates facilitate displacement on faults^49,122–124^. The increasing efficiency of surface processes during the initial phases of rifting results in localizing deformation and increasing fault-block size. Conversely, intermediate to high sedimentation rates over hot extending crustal sectors exert an effect of thermal blanketing that favors viscous/distributed deformation in the basement enhancing the effect of weak crustal rheologies^121^.

## Final remarks and conclusions

This paper, supported by high-quality seismic examples, describes and enhances several key morphological and structural observations to propose a classification organized along two axes reflecting the mechanical behavior of the crust and the magmatic budget while rifting and breakup.

The key observables are the presence or absence of certain domains. Magma-poor margins always present Coupling and Exhumation Domains, and the poorer in magma it is, the longer will be the Exhumation Domain with a very progressive magmatic input. On the contrary, magma-rich margins exhibit a magmatic domain with outer SDR.

Although this classification is a qualitative approach, some of its main propositions may be used quantitively, such as:

- The width of the Necking Domain;
- The initial thickness and width of the Coupling Domain^79^;
- The width of the Exhumation Domain;
- The initial thickness of the outer SDR (or Magmatic) Domain.

Because of a lack of data, we did not consider the conjugates to our examples. The descriptive approach would not change, as asymmetry is not necessarily linked to a certain mechanical behavior^37,105^ and breakup mechanisms are symmetric to slightly asymmetric in terms of domain presence and width. However, having the conjugate margin would give more weight to the quantifiable elements.

It is important to note that rifted margins may suffer several rifting events prior to finally breakup. The multiplicity of events is often related to changes in the external conditions (far field stress evolution, onset or exhaustion of a hotspot, etc.) driving the formation of the rifted margins. In that case, the formation of the rifted margin could either be seen as a whole continuum or limited to the last event that successfully broke the crust up.

## Method and terminology

Our work is based on a descriptive approach of the large-scale correlations, geometries and organization to define a set of observables from multi-channel seismic data. Even if we present a single margin, when available the conjugate has been interpreted jointly. The description of rifted margins is done firstly by defining its vertical layering and its horizontal zonation (domains). Then several key observables are defined.

### Vertical layering

The layering considers the presence and general shape of four layers. They are defined between five key surfaces, from bottom to top:

- The Moho reflection is amongst the deepest reflections visible along a seismic profile. In TWT, it is globally flat and generally around 9 to 11 s (TWT) deep. Its facies and amplitude may vary a lot along a profile. It may be a rather strong and continuous reflector but sometimes it disappears if the velocity contrast is not important enough or even absent (serpentinized mantle or High-Velocity Lower Crustal Bodies for example). It is worth noticing that the Moho interpretation is not necessarily unique. Indeed, some multi-channel seismic lines^125^ exhibit clear reflections within the mantle. These reflections can, in some cases, be continuous and consistent in 3D. Therefore, in areas where it is poorly defined or where there are multiple hypotheses, wide-angle seismic data or simple gravimetric/magnetic inversion may be needed to constrain the location of the Moho;
- Considering that, in this article, the presented margins are all of Mesozoic/Cenozoic age, we chose to locate the Top Basement as the major consistent unconformity at the base of Mesozoic/Cenozoic basins or at the end of the last orogenic event. Therefore, the basement, as it stands here, may be composed of old sedimentary basins (Mesozoic, Paleozoic and older), metamorphosed/strongly folded sediments (from Paleoproterozoic to Cenozoic) or igneous rocks. It is generally easy to identify in the undeformed and proximal domain where it is well imaged and can be directly correlated to onshore geology. However, it is more difficult to interpret it in a rifted margin due to a poorer image quality (important deformation, loss of seismic impedance contrast, etc.). Also, the crust is often separated into sub-layers such as upper, middle and lower crust. In the Oceanic Domain, the Top Basement is the Top of the Oceanic Crust which also coincides with the base of the sediments;
- The Base Rift corresponds to the surface defining the onset of the rifting event. It is often an important unconformity between quite isopachous well-bedded sediments and typical syn-tectonic sequences (fault-controlled wedge-shaped layers). It is merely a matter of definition if layers defined between the Top Basement and the Base Rift, i.e. the Pre-Rift sedimentary unit, which may be composed of several internal layers depending on the geological history of the area (multiple rifting/cooling events) are considered as separate layers or if Top Basement and Base Rift are considered as the same surface, as it is done in this article;
- The Top Rift corresponds to the surface related to the breakup of the lithosphere. It is often named breakup unconformity. In this article, we choose to define the Top Rift as the most important onlap surface (unconformity) coeval with the first clearly observable oceanic crust. Therefore, the Top-Rift coincides laterally to the Top basaltic Oceanic Crust. It is characterized by a drowning or an acceleration of the drowning of the rifted margin recording a tilting associated to large scale subsidence of the margin. The Syn-Rift layer may be composed of several internal layers separated by regional or local unconformities that reflect the migration of the deformation during the rifting. Thus, the Syn-Rift layer represent the whole sedimentary package that registered the stretching, thinning and breakup of the lithosphere. Local or sub-basinal events along the margin are referred as pre-, syn- or post-tectonic sub-layers;
- The Seabed, or topography in onshore domain, defines the top of the Post-Rift sedimentary package. It reflects the balance between the lithospheric cooling following the rifting event and sediment input. This package is represented by wide and parallel reflections onlapping the rifted margin while subsiding. The post-rift package may be locally disturbed by salt and shale tectonics related to large deltaic provinces.

### Rifted margin domains

The morphology of a margin can be translated into its horizontal zonation or domains^13,126,127^. This zonation is based on the relationship between the different layers and horizons defined in Sect. 5.1. and the presence of some characteristic geometries. Different domains can be seen along a rifted margin and are bounded by their onset lines. It is worth noticing that not all domains are observed in all different margins and their presence or absence depends greatly on the context:

- The Proximal Domain is where the crust is barely thinned. Moho reflection, Base-Rift, Top-Rift and Topography/Seabed are globally parallel. The syn-rift deformation is limited. Faulting may be observed in this domain but without any important thinning of the crust. The post-rift subsidence registered here is limited due to a little thinning of the lithospheric mantle during the rifting. This domain ends up at the Necking Line (NL);
- The Necking Domain onsets at the Necking Line (NL) and ends at different lines (Coupling Line, Limit of Oceanic Crust, Limit of Continental Crust) depending on the type of rifted margin and context (see below). The Necking Line corresponds to the onset of crustal thinning and is characterized by a general convergence of Moho reflection with Base-Rift and Top Syn-Rift horizons. In depth section, both Moho and Top-Rift (+ Base Rift) form the typical triangular shape of this domain. In time section, the Moho being relatively flat, Top-Rift strongly dips toward the distal domains;
- The Coupling Line (CL), when existing, is characterized by the first faults cutting the whole crust down to the mantle. They might either root at Moho level or cut down into the mantle. The Coupling Domain onsets at this line and, similarly to the Necking line, ends up at different lines (Limit of Continental Crust or Limit of Oceanic Crust). The Coupling Domain exhibits also a triangular shape with converging Moho and Top Rift (+ Base Rift) horizons. This forms a distal necking accommodated by faults and detachments called Hyper-Extended Continental Wedge (HECW^79^) that deform in a simple shear manner;
- The Limit of Continental Crust (LCC) corresponds to the point where identifiable continental crust elements are no longer visible. The Moho reflection is often lost or blurred and even sometimes doubled, thus geophysical Moho can be different from petrological Moho. Depending on the type of margin and the rapidity of the breakup processes, this limit also corresponds to the onset of either the exhumation domain (mantle exhumation at seafloor), or the Magmatic Domain (outer SDR wedge) or even directly the Oceanic Domain (oceanic crust);
- The Limit of Oceanic Crust (LOC) is defined by the point marking the first recognizable oceanic crust defined by parallel Moho and Top Basement and a thickness of 2 to 2.5 sTWT. In general, the upper seismic facies of the oceanic crust is made of chaotic to relatively flat lying reflections. In some particularly good multichannel seismic data, a lower layered facies and crust-cutting dykes might be observed. The first oceanic crust might form in an ultra-slow setting. Therefore, in term of facies and thickness, it is difficult to differentiate it from the exhumation domain. In that case the LOC is unclear.

### Key observables

Using the layering and the domains of rifted margins previously defined, we can focus secondly on several qualitative observables such as the presence or absence of certain elements or their relative width or thickness. They give key indication on the behavior during rifting and breakup periods. The listed observables are:

- The observed domains. The large variability of rifted margins is illustrated at a first order by the presence or absence of some of the domains previously defined. Proximal and Necking domains are always present. Conversely, Magmatic (or Outer SDR), Coupling and Exhumation domains are not and are characteristic of certain types of margins. The presence of the Magmatic Domain is symptomatic of the magma-rich margin. It forms a distal necking accommodated by magmatic additions. On the other hand, the presence and width of the Exhumation domain is characteristic of magma-poor margins;
- The width of the Necking Domain: this domain is the most variable in terms of width and general shape. This variability is evident looking at worldwide rifted margins^79^ but also at the scale of an ocean (notion of segments). The concept of coupling efficiency^128^ has been used to explain the necking variability along different segments of a single margin (e.g. Norway). The Necking Domain can be very short, even nearly absent, to extremely wide (several hundreds of kilometers). In the case of a very wide Necking Domain, it can be divided into two sub-domains with a primary accentuated necking followed by a long flat to slightly converging taper often composed of several internal sub-basins (neck basins);
- The width of the Coupling Domain: this aspect has been well explored by Nirrengarten et al.^79^. At a first glimpse, the taper angle of the Hyper-Extended Continental Wedge reflects the position of the taper relatively to the main detachment fault: a short taper characterizes the upper plate (above the detachment), a long taper with seaward dipping fault characterizes more generally the lower plate (below the detachment). At a second order, the width of the coupling domain is also dependent on the content of brittle material in the crust. The crustal thinning accommodated in the necking is made in a pure-shear manner and distributed between upper brittle crust and ductile mid to lower crust. Ultimately this ductile material is removed, and only brittle material is preserved in the coupling domain allowing faults to cut through the entire crust;
- The type of structures: The faults accommodating the rifting event can be described considering their shape (high-angle, listric, low-angle, detachment) and their level of rooting within the crust. Their presence and repartition along a rifted margin and their timing are also key to understand the rifting processes;
- The syn-rift (either sedimentary and/or magmatic) infill. The relative thickness of the syn-rift package, its shape, layering and the identification of the period of tectonic activity of a group of faults or a sub-basin of the margin record the deformation and whether it is in-sequence (from distal to proximal) or not.
